# Impact of Tissue Harvesting Sites on the Cellular Behaviors of Adipose-Derived Stem Cells: Implication for Bone Tissue Engineering

**DOI:** 10.1155/2017/2156478

**Published:** 2017-12-14

**Authors:** Maryam Rezai Rad, Mahbobeh Bohloli, Mahshid Akhavan Rahnama, Azadeh Anbarlou, Pantea Nazeman, Arash Khojasteh

**Affiliations:** ^1^Dental Research Center, Research Institute of Dental Sciences, School of Dentistry, Shahid Beheshti University of Medical Sciences, Tehran, Iran; ^2^Department of Tissue Engineering, School of Advanced Technologies in Medicine, Shahid Beheshti University of Medical Sciences, Tehran, Iran; ^3^Department of Applied Cell Sciences, Faculty of Medical Sciences, Tarbiat Modares University, Tehran, Iran

## Abstract

The advantages of adipose-derived stem cells (AdSCs) over bone marrow stem cells (BMSCs), such as being available as a medical waste and less discomfort during harvest, have made them a good alternative instead of BMSCs in tissue engineering. AdSCs from buccal fat pad (BFP), as an easily harvestable and accessible source, have gained interest to be used for bone regeneration in the maxillofacial region. Due to scarcity of data regarding comparative analysis of isolated AdSCs from different parts of the body, we aimed to quantitatively compare the proliferation and osteogenic capabilities of AdSCs from different harvesting sites. In this study, AdSCs were isolated from BFP (BFPdSCs), abdomen (abdomen-derived mesenchymal stem cells (AbdSCs)), and hip (hip-derived mesenchymal stem cells (HdSCs)) from one individual and were compared for surface marker expression, morphology, growth rate, and osteogenic differentiation capability. Among them, BFPdSCs demonstrated the highest proliferation rate with the shortest doubling time and also expressed vascular endothelial markers including CD34 and CD146. Moreover, the expression of osteogenic markers were significantly higher in BFPdSCs. The results of this study suggested that BFPdSCs as an encouraging source of mesenchymal stem cells are to be used for bone tissue engineering.

## 1. Introduction

Application of mesenchymal stem cells (MSCs) for bone tissue engineering has been available for several years [1–6]. However, finding a proper source that is easy to harvest with high cell yield and high potency has been a challenge for researchers. Most of this source was and continues to be autologous bone marrow mesenchymal stem cells (BMSCs) [1–6]. However, the painful tissue collection process, the low cell yield, and the significant age-related differentiation potentials of these cells lead us to search for alternative sources of MSCs as an important aspect considered for regenerative medicine applications [7, 8].

Adipose tissues are an abundant and readily available source, and their harvest procedures are associated with minimal discomfort for the patient [9–11]. Many adipose tissues were discarded following elective liposuctions. Moreover, adipose tissues have the cell yield about 500-fold more than bone marrow aspirates [12, 13]. Also, the isolated cells from adipose tissues have been shown to proliferate rapidly in vitro, demonstrate low levels of senescence after months of in vitro expansion, and have been proven to differentiate toward the osteogenic lineage both in vitro and in vivo [13–15]. Recently, adipose tissue also has been isolated from the buccal fat pad (BFP) [16]. This source of MSCs has gained interest to be used for bone regeneration in the maxillofacial region, since it is easily accessible for dentists and maxillofacial surgeons. The harvesting of BFP is a simple procedure, which requires a minimal incision with local anesthesia and causes minimal donor-site morbidity [16]. The BFP tissues have been used in oral and maxillofacial surgeries including the treatment of congenital oronasal diseases [17], congenital cleft palate repair [18], and intraoral malignant defects [19]. Recent studies showed that adipose-derived stem cells (AdSCs) from the BFP, that is, buccal fat pad-derived stem cells (BFPdSCs), possess all the suitable characteristics for bone tissue engineering, both in vitro and in vivo [20–23].

A few reports have compared the feature of AdSCs isolated from different parts of the body [20, 23]. Farre-Guasch et al. have compared the behavior of human AdSCs from BFP and abdominal subcutaneous fat tissues and they showed that both cells have similar morphology and cell yield. Also, both cells are capable to differentiate into adipogenic, osteogenic, and chondrogenic lineages [20]. Niada et al. conducted an experiment on porcine AdSCs from BFP and subcutaneous interscapular site and they showed no difference in proliferation, viability, and clonogenicity. Also, both types of cells demonstrated osteogenic differentiation capability [23]. However, a study by Broccaioli et al. on human BFPdSCs and AdSCs from abdominal tissues (AbdSCs) showed that AbdSCs proliferate more rapidly. They also showed that these cells differentiated towards the osteoblastic lineage similarly; however, the expression of ALP markers were different in them [24]. The higher level of ALP activity was observed in AdSCs harvested from BFP. However, the collagen production were significantly higher in AbdSCs [24].

Due to the scarcity of the data regarding comparative analysis of isolated AdSCs from different parts of the body and considering the high potential of AdSCs for cell therapy in bone regeneration, there is a certain need to quantitatively compare the osteogenic capability of AdSCs from different sites. Therefore, in this study, we sought to compare AdSCs from different parts of the body, including AbdSCs, BFPdSCs, and hip-derived mesenchymal stem cells (HdSCs). Since the donor variability would make the comparison internally consistent, cells derived from same donors have been compared for the following: (1) surface marker expression, (2) morphology, (3) growth rate, and (4) osteogenic differentiation capacity in a quantitative manner.

## 2. Materials and Methods

### 2.1. Adipose Tissue Collection and Isolation of MSCs

Adipose tissues were obtained from three healthy volunteer donors (two males and one females) with the age range of 25–35 years with informed consent. The study was approved by the Ethics Committee of Shahid Beheshti University of Medical Sciences. In each patient, the same amount of tissue volume, that is, 5 ml, was harvested from abdomen, hip, and BFP. Harvested autogenous tissues were immediately processed using enzymatic digestion using the protocols explained in previous studies [20, 23, 24]. Briefly, the tissues were washed with phosphate-buffered saline (PBS) (Sigma-Aldrich, St. Louis, Missouri, United States) twice. Then, they were digested in a solution of 3 mg/ml type I collagenase (Sigma-Aldrich, St. Louis, Missouri, United States) for 30 min at 37°C followed by centrifugation. Resultant pellet was termed as stromal vascular fraction (SVF) by Federation of Adipose Therapeutics and Sciences (IFATS) [25] which consists of not only adipose stromal and hematopoietic stem cells but also endothelial cells, erythrocytes, fibroblasts, lymphocytes, monocyte/macrophages, and pericytes. Then, the SVF cells were resuspended in stem cell growth medium containing Dulbecco's modified Eagle's medium high glucose (DMEM-HG) (Life Technologies, California, United States) supplemented with 15% fetal bovine serum (FBS) (Life Technologies, California, United States) and 1% penicillin/streptomycin (Life Technologies, California, United States) and cultured in T-25 flasks under 5% CO_2_ at 37°C. No specific selection was used. Instead, these cells were furthered purified using a combination of washing steps and culture expansion with the given medium. This process depletes most of the hematopoietic cell population from the SVF cells and allows the emergence of an adherent cell population termed as AdSCs. The cells were passaged at 90% confluency using 0.25% trypsin-EDTA (Life Technologies, California, United States) at a ratio of 1 : 3 until P2 was obtained. Since different proliferation rates of AdSCs from various tissues were observed, the cells at P2 were cryopreserved in 10% DMSO (Sigma-Aldrich, St. Louis, Missouri, United States) when they reached 90% confluency. So subsequent experiments were started at the same time for all three types of cells.

### 2.2. Characterization of AdSCs

First, the AdSCs, that is, AbdSCs, BFPdSCs, and HdSCs, were evaluated for cell surface marker expression. Three types of cells at passage 3 (P3) were harvested using 0.05% trypsin-EDTA. The harvested cells were resuspended in PBS at a concentration of 10^5^ per sample stained for 30 min at 4°C in the dark room with antibodies against human CD34-FITC, 581 (EXBio, Vestec, Czech Republic), CD44-FITC, MEM-263 (EXBio, Vestec, Czech Republic), CD45-FITC, H130 (BD Biosciences, San Jose, CA, USA), CD73-FITC, E12169 (e Biosciences, San Diego, CA, USA), CD90-FITC (CMG, Esfahan, IRAN), CD105-PE, MEM-226 (EXBio, Vestec, Czech Republic), and CD146-PE, P1H12 (BD Biosciences, San Jose, CA, USA) at a concentration of 2 *μ*g/ml. After incubation time, cells were washed with PBS. Analysis was next performed using flow cytometer.

Moreover, the cells at P3 were assessed for multilineage potential towards the adipocytes, osteoblasts, and chondroblasts. To do this, the cells were cultured in 24-well plates, at a concentration of 10^4^ cells/well, in induction medium. For osteogenesis, cells were cultured for 14 days in Stem Pro (Life Technologies, California, United States) induction medium. Alizarin Red staining (Sigma-Aldrich, St. Louis, Missouri, United States) was administered to assess the mineral deposition following induction. For adipogenesis, cells were cultured for 14 days in adipogenic medium (Life Technologies, California, United States). Following induction periods, cells were then stained with Oil Red O (Sigma-Aldrich, St. Louis, Missouri, United States) to evaluate adipogenesis. For chondrogenic induction, the cells were subjected to chondrogenic medium (Life Technologies, California, United States). Chondrogenic differentiation was assessed by toluidine blue staining (Sigma-Aldrich, St. Louis, Missouri, United States) after 14 days. The stained cells were imaged using inverted light microscopy. The cells cultured in standard growth medium were also imaged after 5 days for morphology assessment.

### 2.3. Evaluation of Proliferation Capacity of AdSCs

The growth characteristics of AbdSCs, BFPdSCs, and HdSCs were compared through long-term culture condition (P3–P8). The cells were cultured in 24-well plates at a density of 10^4^ cells/well; the expanded cells were analyzed and counted using the trypan blue exclusion method and a hemocytometer [26]. In each passage, the cells were cultured for 5 days, then they were detached using 0.25% trypsin-EDTA, counted, and reseeded. This procedure was repeated until the cells reached P8. The population doubling time (PDT) was also examined for each passage using the following formula: (*t* − *t*_0_) · log_2_/log(*N* − *N*_0_), where *t* − *t*_0_ is culture period based on hour, *N* is the number of detached cells, and *N*_0_ is the number of seeded cells.

### 2.4. Evaluation of Osteogenic Capability of AdSCs

The osteogenic differentiation capability of AbdSCs, BFPdSCs, and HdSCs was also determined by evaluation of osteogenic markers using real-time RT-PCR. Primers used for real-time RT-PCR are listed in Table 1. In order to avoid the possible effect of cell number on osteogenic capability of these three populations, the cells were cultured in 6-well plates, at a concentration of 10^5^ cells/well, which are the adequate number of the cells to start the osteogenic induction, without any further need for propagation. After 24 h, the cells were subjected to osteogenic medium for 7 and 14 days. The controls were the cells harvested after 24 hours in standard medium. The expression of alkaline phosphatase (ALP), bone morphogenetic protein 2 (BMP2), collagen type I (COLI), secreted phosphoprotein 1(SPP1), and Runt-related transcription factor 2 (RUNX2), was evaluated as follows. First, the cells were dissociated with Tryzol reagent followed by RNA isolation using precipitation technique [27]. Then, the quantity and quality of isolated RNA was determined using Nanodrop spectrometer. RNA was then reverse-transcribed using cDNA kit (Thermo Scientific, Massachusetts, United States). cDNA templates were used for SYBR Green real-time PCR to detect cycle threshold (CT) values with Applied Biosystems' Real-Time PCR System. The CT values were normalized to *β*-actin to calculate ΔCT. RGE was calculated with the formula 2^−ΔΔCT^ using the control, that is, cells cultured in standard culture medium, as the reference (RGE = 1).

Osteogenic capability of the given cells was also compared at the protein level. For ALP activity assay [28], total protein of the cells was extracted using radio immunoprecipitation assay (RIPA) (Sigma-Aldrich, St. Louis, Missouri, United States) lysis buffer. Then, ALP activity was measured using ALP assay kit (Sigma-Aldrich, St. Louis, Missouri, United States). Absorbance was measured at 405 nm with ELISA reader. For evaluation of BMP2 protein, the medium were collected from the three populations after 7 and 14 days. Then, BMP2 concentration was evaluated using Human BMP2 ELISA Kit [29] (Sigma-Aldrich, St. Louis, Missouri, United States). Absorbance was measured at 450 nm with ELISA reader.

### 2.5. Statistical Analysis of Data

All experiments were repeated three times, and three replications are biological replications, that is, adipose tissues were obtained from three healthy volunteer donors. Quantitative data were expressed as mean ± standard deviation (SD). One-way analysis of variance (ANOVA), followed by Tukey's test at a significance level of *P* ≤ 0.05 was used for the comparison of multiple sample means.

## 3. Results

### 3.1. Comparison of Characterization of AdSCs

**Figure 1:**
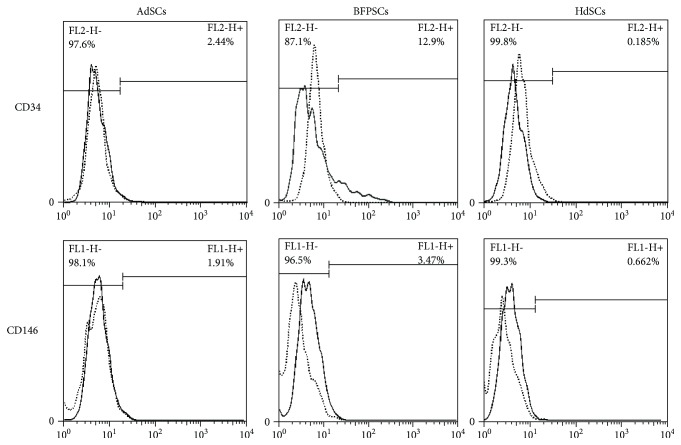
The flow cytometry histograms of CD34 and CD146 from AbSCs, BFPdSCs, and HdSCs.

The flow cytometer analysis revealed that AbSCs, BFPdSCs, and HdSCs expressed MSC-defined markers including CD44, CD73, CD90, and CD105, but they were negative for CD34 and CD45 (Table 2). Among them, BFPdSCs showed some expression of CD34 and CD146, that is, 12.9% and 3.46%, respectively, suggesting a small population of CD146- and CD34-positive population in the BFPdSCs. The histograms for expression of CD34 and CD146 are shown in Figure 1.

**Figure 2:**
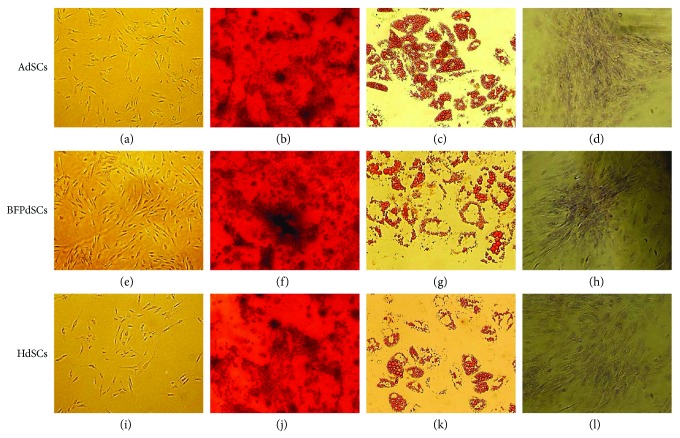
Multilineage differentiation capability of adipose-derived MSCs: (a–d) AbSCs, (e–h) BFPdSCs, and (i–l) HdSCs in various culture conditions including stem cell growth medium (a, e, i), osteogenic induction medium (b, f, j), adipogenic induction medium (c, g, k), and chondrogenic induction medium (d, h, l). Note that all three types of cells displayed a spindle-like morphology. Also, note that multilineage differentiation capability of all three given cells was confirmed by Alizarin Red staining (b, f, j), Oil Red O (c, g, k), and toluidine blue staining (d, h, l).

The evaluation of multilineage differentiation capability showed that all three AdSCs are multipotent and they differentiate towards the osteogenic, adipogenic, and chondrogenic lineages in the presence of inductive medium (Figure 2).

Moreover, AbSCs, BFPdSCs, and HdSCs showed similar morphology and all three exhibited a spindle-shaped morphology (Figure 2, a, e, i).

### 3.2. Comparison of Proliferation Capacity of AdSCs

**Figure 3:**
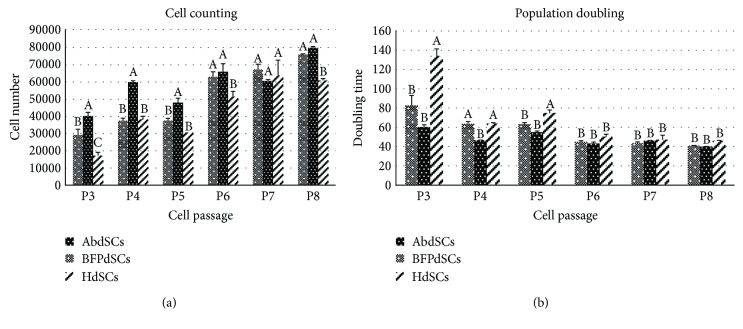
Proliferation rate of adipose-derived MSCs: (a) The cell growth rates of AbSCs, BFPdSCs, and HdSCs at different passages (P3–P8) as determined by trypan blue staining for detection of viable cells. Note that the highest proliferation rate was related to BFPdSCs. (b) Population doubling time of given cells at different passages (P3–P8) after 5 days with the initial density of 10^4^ cells/well. Note that the shortest doubling time was observed for BFPdSCs. Different letters indicate significant difference at *P* ≤ 0.05.

The proliferation rate of all AdSCs were evaluated form P3 to P8 (Figure 3(a)). From P3–P5, BFPdSCs were shown to proliferate significantly faster compared to AbSCs and HdSCs. However, at higher passages, that is, P6-P8, all three AdSCs showed almost similar growth rates (Figure 3(a)). PDT was also evaluated for every passage until P8 (Figure 3(b)). At lower passages (P3–P5), the shortest doubling time was observed at BFPdSCs. Whereas, from P6 to P8, the PDT was similar for all the given cells. In general, the lowest proliferation rate and the highest doubling time were related to HdSCs.

### 3.3. Comparison of Osteogenic Capability of AdSCs

**Figure 4:**
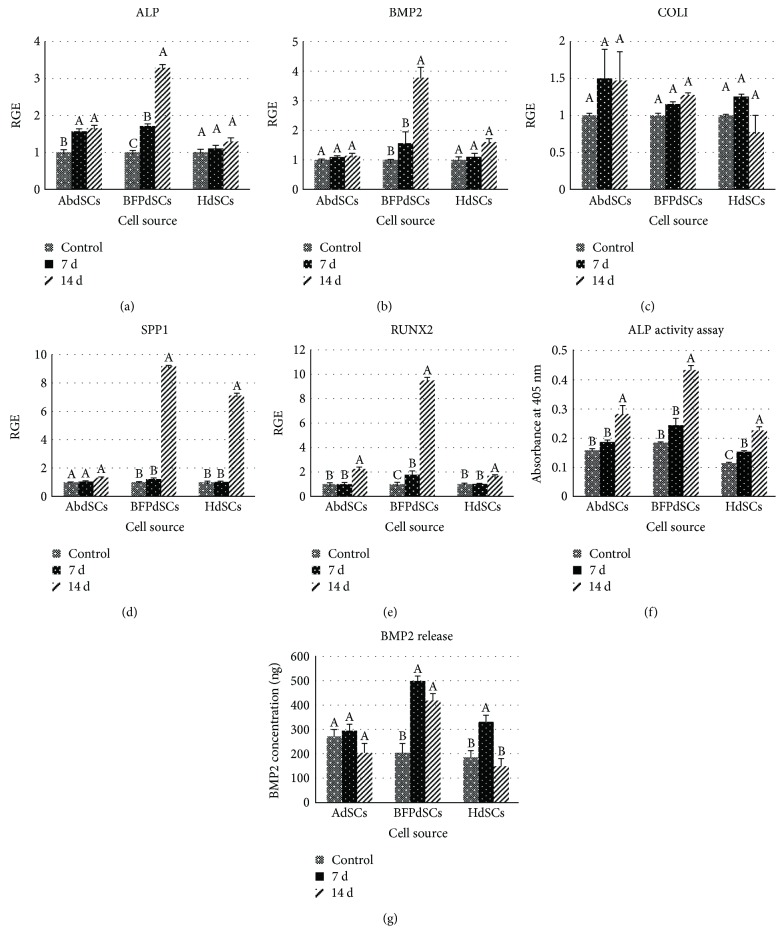
Osteogenic capability of adipose-derived MSCs: (a–e) Relative gene expression (RGE) of osteogenic genes (ALP, BMP2, COLI, SPP1, and RUNX2) of AbSCs, BFPdSCs, and HdSCs after 7 and 14 days in osteogenic induction medium versus the cells grown in stem cell growth medium. (f) The expression of ALP at protein level using ALP activity assay. (g) The expression of BMP2 at protein level using BMP2 ELISA kit. The amount of ALP and BMP2 were the highest in BFPdSCs at both day 7 and 14 of osteogenic induction. Different letters indicate significant difference at *P* ≤ 0.05.

The expression of both early osteogenic-related markers, that is, ALP, BMP2, and COLI, and late osteogenic markers, that is, SPP1 and RUNX2, were evaluated following 7 and 14 days of osteogenic induction (Figure 4). The expression of the given genes in the cells grown in stem cell growth medium was considered the basal level of expression and used as a reference point for comparison. The expression of the early markers, ALP and BMP2, were significantly higher at BFPdSCs at both 7 and 14 days (Figures 4(a) and 4(b)). However, no significant difference was observed in the expression of COLI between AbSCs, BFPdSCs, and HdSCs at both time points (Figure 4(c)). The later osteogenic markers, SPP1 and RUNX2, were expressed similarly in all three AdSCs at day 7th of induction, whereas the expression of this gene was significantly higher at BFPdSCs at day 14th (Figures 4(d) and 4(e)).

The expression of ALP and BMP2 at translational level showed that, among them, the ALP activity as well as expression of BMP2 in BFPdSCs was significantly higher after 7 and 14 days of osteogenic induction (Figures 4(f) and 4(g)).

## 4. Discussion

An ideal cell source should have the following characteristics: (1) available easily, (2) harvest with minimal discomfort and morbidity for the patient, (3) have adequate cell yield, (4) process easily with minimal cost, (5) have high proliferation and differentiation capabilities, (6) have potency not related to patientsʼ age and gender, (7) maintain their proliferation and differentiation capabilities after culture expansion, and (8) have immunomodulatory effect which make them suitable for allografting [11]. Despite the application of MSCs in bone tissue engineering for several years [1–6], finding a cell source, having all given characteristics, still is challenging.

AdSCs have been used as an alternative to BMSCs since they are available in large quantities as a result of patients' elective cosmetic surgeries. Moreover, adipose tissues yield higher numbers of MSCs than bone marrow aspirates, which could avoid cell expansion. Also, it has been shown that their differentiation capability is not age related [9–13].

The oral cavity also has a specific fatty tissue, BFP, which is different from other subcutaneous fat tissues [30]. This tissue is an easily accessible source in craniofacial region which has been gaining attention from dentists and maxillofacial surgeons. Moreover, harvesting of BFP tissue is not a complicated procedure and is performed by means of a minimal intraoral incision with local anesthesia with minimal donor-site morbidity [16]. Also, in contract to other subcutaneous tissues, the size of BFP is similar among different patients and several studies have demonstrated that BFP size is not related to the patient's body weight and fat distribution. It means patients having a lower weight with little subcutaneous fat have similar BFP compared to patient with the normal weight [20, 31].

Besides, it is well known that adipose tissues are derived from various embryonic origins [32]; for example, adipose tissue of the craniofacial region is originated from the neural crest [33] and adipose tissue in other parts of the human body raised from other embryonic tissues [34]. Considering the fact that majority of craniofacial tissue originates from neural crest, application of MSCs derived from a nearby site of the defect with similar embryonic origin may further enhance regenerative outcomes [23, 35]. This fact makes the BFPdSCs a proper source for bone regeneration in craniofacial region.

A few studies have evaluated the effect of human adipose tissue-harvesting sites on biological behaviors of AdSCs [20, 23]. However, in these studies, the adipose tissues were harvested from variable donors which make the comparison difficult. Also, in order to use MSCs derived from adipose tissue for bone regeneration, we need to compare their osteogenic capability in a quantitative manner. Hence, in the current study, we compared AdSCs from three different body areas, that is, abdomen, BFP, and hip in terms of their morphology, proliferation rate, MSC surface marker expression, and expression of osteogenic marker genes.

All three types of AdSCs were successfully isolated from harvested tissues and presented similar fibroblast-like morphology. Also, their multilineage differentiation capabilities towards the adipocytes, osteoblasts, and chondroblasts were confirmed.

In this study, we have compared the growth kinetics of AdSCs at different passages. We showed that BFPdSCs proliferate significantly faster with the shortest doubling time at lower passages (P3–P5). However, this difference was not significant from P6 to P8. Several studies have demonstrated the effect of culture expansion on differentiation capability of MSCs [36–38]. Also, the risk of genetic alteration, oncogenesis, cellular senescence, and bacterial contamination will be increased during the cell culture expansion [39–42]. Hence, the high proliferation rate of BFPdSCs at lower passages, which are considered safe and proper passages for therapeutic applications, would make them suitable for clinical applications. In contrast to our finding, a study by Niada et al. showed that AdSCs from abdominal subcutaneous site had faster proliferation rate and shorter doubling time [23]. This difference might be due to the fact that they harvested the tissue from different donors at different ages which were termed as an intrinsic characteristics of the patients [43].

The expression of MSC markers including CD44, CD45, CD73, CD90, and CD105 were similar in AbSCs, BFPdSCs, and HdSCs and it was in according to the International Society for Cellular Therapy [44]. However, among those, BFPdSCs showed higher expression of CD34 and CD146.

CD34+ cells have been shown capability of stimulating angiogenesis, and they are involved in neovascularization processes [45, 46]. CD146 is a marker of endothelial progenitor cells, which are found in the rich microvasculature within this adipose tissue [47]. Also, it has been shown that there is a MSC population, termed as perivascular stem cells, found in vascularized tissues, consisting of CD34+ and CD146+ cells [47, 48]. Studies demonstrated that they not only are involved in angiogenesis, but they also display high osteogenic potential [49, 50] which make them attractive for vascularized bone regeneration.

The presence of population of CD146+ and CD34+ cells in BFPdSCs might be due to the existence of highly enriched blood vessel supply in BFP [16]. Considering the importance of angiogenesis in bone regeneration, the existence of given population would make BFPdSCs a proper candidate for cell therapy in maxillofacial bone regeneration. Moreover, we showed a higher osteogenic potential in BFPdSCs comparing to AdSCs from abdomen and hip. This might be attributed to the higher proliferation rate of CD146+ and CD34+ cells within the BFPdSC population.

Finally, for bone tissue engineering, comparative analysis of the expression of osteogenic related genes are essential. We have shown that the early osteogenic markers including ALP, both at mRNA level and protein level, and BMP2 were significantly upregulated in all three types of the cells. However, this expression was significantly higher in BFPdSCs. The expression of COLI, another early marker of osteogenesis, was similar among the three cell types. A study by Niada et al. also compared the expression of ALP and COLI in AdSCs from BFP and abdomen. In agreement to our findings, they have shown that the ALP activity was significantly higher in AdSCs from BFP. However, they showed that production of COLI was significantly higher in AdSCs of the abdomen [23]. Moreover, we have compared the expression of osteogenic later markers, RUNX2 and SPP1. Significant expression of these genes, especially at a later stage of osteogenesis, for example, on the day 14th of induction, was considerable in BFPdSCs. Despite the limited amount of tissue available in BFP comparing to abdomen and hip, greater expression of osteogenic genes in BFPdSCs at both early and later stages of osteogenic differentiation would suggest them as a suitable source for bone regeneration.

In conclusion, the higher proliferation rate and the high osteogenic capability and expression of markers relevant to angiogenesis make BFPdSCs an encouraging source of MSCs to be used for bone tissue engineering. Although the proliferation rate and capacity of osteogenic differentiation are important parameters for selection of a cell source for cell-based therapies of the bone, the evaluation of BFPdSCs for immunomodulatory effect would be beneficial in order to consider this valuable source of MSCs for allografting as well.

## Figures and Tables

**Table 1:** Primer used for real time RT-PCR.

Gene name	Primer	Sequence 5′ to 3′
ALP	Forward	CGGAACTCCTGACCCTTGAC
	Reverse	ATTCTGCCTCCTTCCACCAG
BMP2	Forward	GGACGCTCTTTCAATGGACG
	Reverse	AGCAGCAACGCTAGAAGACAG
COLI	Forward	TGGAGCAAGAGGCGAGAG
	Reverse	CACCAGCATCACCCTTAGC
SPP1	Forward	GACCTGACATCCAGTACCCTG
	Reverse	GTGGGTTTCAGCACTCTGGT
RUNX2	Forward	GAACCCAGAAGGCACAGACA
	Reverse	ACTTGGTGCAGAGTTCAGGG
Actin	Forward	ATGCCTGCCGTGTGAAC
	Reverse	ATCTTCAAACCTCCATGATG

**Table 2:** The expression of cell surface markers.

	CD34	CD44	CD45	CD73	CD90	CD105	CD146
AbdSCs	2.44%	93.90%	0.65%	99.50%	99.60%	99.40%	1.91%
BFPdSCs	12.90%	95.50%	1.70%	96.40%	99.30%	99.20%	3.47%
HdSCs	0.19%	91.10%	0.65%	99.40%	99.40%	99.20%	0.66%

Data are the average of 3 replicates, that is, donors.
